# Special Departments

**Published:** 1910-09-03

**Authors:** 


### SPECIAL DEPARTMENTS.

### MIDWIFERY—OPHTHALMOLOGY—DERMATOLOGY—LARYNGOLOGY.

The special departments of a hospital, while not exacting from the student the same amount of attention as the general wards, are nevertheless very necessary to the proper education of one whose aim should be the acquirement of an all-round knowledge such as befits a competent general practitioner. The mere fact that it is possible with luck to pass the final examination with but the scantiest knowledge of diseases connected with the eye, ear, throat, nose, skin, etc. should not encourage the student to neglect these departments. On the other hand, it would be equally foolish for him to specialise in one or more of these subjects at the expense of his general knowledge. His object should be the attainment of a happy medium between these two extremes—that is, a knowledge of the more common diseases met with in the practice of each department, and of their bearing on general medicine and surgery.

#### Gynæcology and Obstetrics.

The department connected with the study of midwifery and the diseases peculiar to women is, however, an exception to this general rule. The attendance of the student here is compulsory, and he will be obliged to produce evidence that his work has been both adequate and satisfactory. Some of the higher examining bodies also insist on a course of training for a period of three months in the wards attached to the obstetric department. This requirement is not, however, universal, but the keen student will take advantage of any opportunity afforded of entering the wards, even though the regulations concerning the particular diploma or degree he contemplates entering for do not insist upon his doing so. The bedside training so obtained can in no way be replaced by even the most diligent attendance at the out-patient department. The progress made by a case can be followed up, and methods of treatment learnt in a manner quite different to that afforded by the necessarily somewhat irregular attendance of patients who only come up for observation as out-patients. The student will stand less in need of any hints we may give as regards this than with reference to some of the other special departments, since the very fact that a separate examination has to be passed in the subjects connected with it will ensure that he realises its importance.

#### Diseases of the Eye.

We therefore pass on to the departments which stand next in importance, namely those connected with diseases of the eye and of the skin. Of these the first mentioned should perhaps attract the most attention. The eye is a very delicate organ, and failure to recognise a diseased condition in it may lead to loss of sight or even of the organ itself. The student should, therefore, pay particular attention to eye injuries and endeavour to acquire a rough knowledge of the gross changes occurring in the fundus as the result of disease. To examine the latter it is necessary to use the ophthalmoscope, an instrument which is daily becoming more important in general medicine, and a knowledge of the use of which is demanded in most University examinations. Every student should obtain an ophthalmoscope as soon as he enters the medical wards, and make a practice of using it on every possible occasion. He may, and probably will, find considerable difficulty at first in seeing anything with it. Constant practice and the attendance at lectures and demonstrations on its use, provided by the ophthalmic department of every teaching hospital, will soon overcome this difficulty. There are several patterns of instrument on the market, but on the whole that named after Morton is the most satisfactory, and will repay the slightly greater outlay required for its acquisition. Opportunities, however, arise at times of obtaining a satisfactory instrument secondhand from a newly-qualified man who, for some reason or other, wishes to dispose of his. The student who can afford it should also purchase a loupe. This instrument is almost essential for the proper examination of superficial injuries to the cornea, sclerotic, etc., as well as for appreciating the existence of morbid conditions of that membrane, such as keratitis punctata. Again, if his time will allow of it he should endeavour to obtain some knowledge of refractions. Skill in this branch of ophthalmic practice is in some cases a considerable source of income to the country practitioner. It is well to possess some text-book on the eye, as, with few exceptions, the ordinary surgical manuals do not deal thoroughly with diseases of that organ. The excellent little book by J. Hutchinson, junior, entitled "Aids to Ophthalmic Medicine,"* deals concisely with all the commoner affections of the eye.

The contents of this or some similar work should be mastered before proceeding to read larger books.

Dermatology.

Diseases of the skin cannot be learnt from text-books, neither can a true idea of their characteristics be obtained from the perusal of even the most elaborate plates. Attendance in the skin department is therefore an absolute necessity. The diagnosis of skin lesions is by no means easy, and long practice and the study of a great number of cases will be necessary before the student will feel any confidence in his powers of distinguishing between the numerous morbid conditions to which the skin is subject. He will, however, be amply repaid in later life for any extra labour which his studies may have entailed, not to mention the fact that skin cases are at times met with in examinations. Having once seen a case of skin disease it is well to read up all about it in a text-book, as the description of a skin lesion is not an easy matter, and accuracy of expression is very necessary in medical matters. Reference to a reliable medical vocabulary or dictionary of medical terms will explain any expressions with which the student is unacquainted. The language of the dermatologist is full of unfamiliar words.

The Throat and Nose.

The throat department comes next in importance. In many hospitals diseases of the nose and throat are treated in conjunction with those of the ear. In some, however, this last has a department to itself. Whichever be the case, there is always a considerable amount of overlapping, for in many patients diseases of two or more of these organs are coexistent, or the disease may extend from one to the other. It is in the throat department that opportunities will arise for the student to perfect himself in the use of the laryngoscope. This again is an instrument which is at first by no means easy to manipulate. Every chance of seeing it in use and of using it personally should therefore be taken. Much can be learnt from watching an expert at work. The proper method of examining a nose should also be acquired. The student should endeavour to be present at operations on the throat and nose, and perhaps more especially at such simple ones as those for the removal of tonsils and adenoids. It is the simpler and more common operations that he will be called upon later to perform, either as a house surgeon or as a general practitioner. An exaggerated importance is apt to be attached to the seeing of major operations, and students will flock to the theatre to view a surgeon performing some rare and difficult operation such as they themselves will never be called upon to carry out, while neglecting to be present at the minor operations which will form a large part of their future surgical practice.

The Ear and the Teeth.

The ear department will furnish material for the acquisition of practice, in the use of the ear speculum, another instrument which demands patience on the part of the user before its advantages can be appreciated. The proper method of manipulating an aural syringe for the removal of wax from the ears is also to be learnt. At first sight the dental department might not seem to have much connection with the work of a medical student, but it must be remembered that a doctor, especially in the country, will often be called upon to extract a tooth. Practice alone will ensure the student being able to perform in a satisfactory manner this apparently simple operation. The dental surgeon attached to the hospital will give many useful hints both as to the proper instrument to use for each type of tooth, as well as to the correct method of approaching the patient and applying the instrument so as to avoid breaking the tooth. It is therefore by no means a waste of time to employ a vacant half-hour in attending a dental clinique occasionally. Moreover, it has of late become more and more recognised that there is a good deal of connection between various digestive disturbances and the presence of dental caries, pyorrhœa, etc., in a patient. Some surgeons even go so far as to connect obscure joint inflammations with dental defects. A septic mouth would in some cases seem to determine the onset of certain inflammatory conditions of the eye. The student should therefore learn to recognise pyorrhœa alveolaris, dental caries, both active and arrested, and the other ordinary diseased conditions of the teeth and mouth, and he can have no better opportunities of so doing than are met with in the average dental department of a general hospital.

Other Departments.

The department devoted to electrical and physical therapeutics, including the *x*-rays, Finsen light, etc., comes more into the province of the post-graduate than of the ordinary student. It is well, however, to acquire at least an elementary knowledge of the working of the more common instruments, such as light-baths, high-frequency apparatus, various batteries and induction coils, etc., etc. The opportunity should not be neglected of seeing fractures through the fluorescent screen of the *x*-ray apparatus, for it is not always as easy as it may seem to locate a simple linear fracture with the eye. A skiagram has often revealed the presence of a fracture which has been missed at a screen examination. The appearances of bones as seen in a skiagram should also be studied. The normal will often appear abnormal to those unused to the reading of radiographic shadows. Much help in this matter can be obtained from the radiologist in charge of the department, who will be found always ready to put his knowledge at the disposal of the keen student. We have thus dealt with the special departments such as are found at most of the general hospitals. In some, however, in addition to those mentioned above, special departments are devoted to orthopædics and diseases of the urogenitary system and rectum. Both are, of course, special branches of general surgery. The last-mentioned is of great value to the post-graduate, but of less importance to the student. It would be as well for the latter, however, to have some knowledge of the working of the urethroscope, protoscope, and cystoscope, instruments used in this department for the internal examination of some of the hollow viscera.

* London : Baillière, Tindall and Cox. Price 2s.

